# Trajectories of mental health in children and adolescents during the COVID-19 pandemic: findings from the longitudinal COPSY study

**DOI:** 10.1186/s13034-024-00776-2

**Published:** 2024-07-18

**Authors:** Anne Kaman, Janine Devine, Markus Antonius Wirtz, Michael Erhart, Maren Boecker, Ann-Kathrin Napp, Franziska Reiss, Fionna Zoellner, Ulrike Ravens-Sieberer

**Affiliations:** 1 Department of Child and Adolescent Psychiatry, Psychotherapy, and Psychosomatics, University Medical Center Hamburg-Eppendorf, Hamburg, Germany; 2 Department of Research Methods, Freiburg University of Education, Freiburg im Breisgau, Germany; 3 Alice Salomon University of Applied Science, Berlin, Germany; 4 Child Neuropsychology Section, Department of Child and Adolescent Psychiatry, University Hospital, Aachen, Germany; 5 Institute of Medical Psychology and Medical Sociology, University Hospital of RWTH Aachen University, Aachen, Germany

**Keywords:** Quality of life, Externalising mental health problems, Internalising mental health problems, Psychosomatic symptoms, Youths

## Abstract

**Background:**

Mental health and health-related quality of life (HRQoL) in children and adolescents deteriorated during the COVID-19 pandemic. The aim of this population-based longitudinal study was to explore whether distinct mental health trajectories in youths can be identified over the course of the pandemic.

**Methods:**

Mental health problems (MHP), psychosomatic symptoms and HRQoL were assessed at five time points between May 2020 and October 2022 in 744 children and adolescents aged 7 to 20 years using established instruments. We used generalized mixture modeling to identify distinct mental health trajectories and fixed-effects regressions to analyse covariates of the identified profiles of change.

**Results:**

We found five distinct linear latent trajectory classes each for externalising MHP and psychosomatic symptoms and four trajectory classes for internalising MHP. For HRQoL, a single-class solution that indicates a common development process proved to be optimal. The largest groups remained almost stable at a low internalising and externalising symptom level (64 to 74%) and consistently showed moderate psychosomatic symptoms (79%), while 2 to 18% showed improvements across the pandemic. About 10% of the youths had consistently high internalising problems, while externalising problems deteriorated in 18% of youths. Class membership was significantly associated with initial HRQoL, parental and child burden, personal resources, family climate and social support.

**Conclusions:**

The mental health of most children and adolescents remained resilient throughout the pandemic. However, a sizeable number of youths had consistently poor or deteriorating mental health. Those children and adolescents need special attention in schools and mental health care.

**Supplementary Information:**

The online version contains supplementary material available at 10.1186/s13034-024-00776-2.

## Background

There is strong empirical evidence that the COVID-19 pandemic has posed substantial mental health challenges to children and adolescents. Systematic reviews and international surveys report considerable impairments of children’s and adolescents’ mental health during the pandemic [1–6]. However, some single studies also show mixed results depending on the time of assessment, instruments, country and study design [6, 7]. Data suggests that both COVID infection dynamics and protection measures are positively associated with psychopathology [6, 8–10]. Studies showing a deterioration of youth mental health mainly focus on the first 1.5 years of the pandemic [5, 6]. Most of those studies compare the pre-pandemic mental health with that during the pandemic or report two measurement points during the pandemic [7], but longitudinal studies are still scarce.

To our knowledge, the German nationwide longitudinal COPSY (COvid-19 and PSYchological Health) study is the first study exploring the mental health of children and adolescents across a time span of three pandemic years. In year one of the pandemic, general mental health problems, anxiety and depressive symptoms as well as the number of children with a low health-related quality of life (HRQoL) have been elevated compared to pre-pandemic data [11]. In year two, mental health stabilised on an impaired level [12], while in year three an improvement became visible for HRQoL, general mental health problems and anxiety. Depressive symptoms even recovered to prepandemic levels [11]. To our knowledge, there are hardly any comparable large-scale longitudinal published studies on the mental health of children and adolescents during the second and third year of the pandemic.

When reading the plentitude of studies published so far, it is surprising that most studies only report average results of their studied population. However, there is no empirical evidence on whether there are groups of children and adolescents who reacted similarly or differently to the pandemic challenges during the pandemic. Generalized mixture modeling (GMM) can be used to identify subgroups of participants with similar patterns of mental health trajectories. According to a recent review of studies, latent class analyses (LCA) and GMM are getting more common in child mental health research [13]. However, we only found one paediatric study [14], which identified different mental health groups during the COVID-19 pandemic. Wang et al. [14] examined 2352 adolescents in China and found a resistance vs. a dysfunction group based on depressive and anxiety symptoms using latent growth mixture modeling of longitudinal data of three waves. Reviews of studies using GMM in adult data [15–16] summarise studies before and during the pandemic and report that approximately 66% of stress-exposed individuals respond resiliently, 13–21% show recovery responses, 9–12% show delayed mental distress, and about 11% report chronic distress in the face of general (and pandemic) stress.

In the present study, we aim to go a step beyond the previous statistics reported in most youth studies, which assume that the development in the study population follows a uniform model of change. We want to learn more about different mental health trajectories as well as potential predictors being associated with those trajectory groups. This allows a detailed characterisation of the development, which is essential to guide and target mental health prevention and intervention programs. We are particularly interested in exploring whether different group trajectories exist for internalising and externalising mental health problems and HRQoL because those are central constructs of positive and negative mental health, which have been heavily overlooked at the start of the pandemic and have been significantly impaired in youth during the pandemic. In the COPSY study those constructs were also chosen to allow comparisons to pre-pandemic studies. Further, previous studies showed that psychosomatic symptoms also increased during the pandemic but were widely overlooked and under-researched. Thus, we also added psychosomatic symptoms as a main construct to our analyses.

This study extends the previous analytical approach of the COPSY study, which was limited to the analysis of covariates of change. To understand potential predictors of trajectory groups of mental health, HRQoL and psychosomatic symptoms, we selected a variety of sociodemographic, personal, familial, and social factors. Previous publications found a gender effect in that girls showed lower HRQoL and more anxiety, depressive and psychosomatic symptoms during the pandemic than boys, while boys had more externalising mental health problems like hyperactivity and conduct behavior [11]. Further, age effects were found in previous studies in that older females reported more anxiety [11]. Those tendencies are in line with a recent review [6]. Our COPSY study so far also found that youths with a good family climate, social support and personal resources were quite resilient [12, 17] and other studies showed fewer negative effects or even benefits during the pandemic [18–20]. Moreover, children may also have benefited from the pandemic, as they were able to spend more quality time with their parents during the lockdowns and may have avoided school and social problems [21, 22]. Further, the COPSY study identified important risk factors for child mental health and HRQoL. These include having a parent with a mental health problem or a parent that felt burdened by the pandemic, a migration background, low parental education and limited living space [11]. Based on these previous research findings and their significance in the context of the study, gender, age, parental education, migration background, single parenting, parental pandemic burden, parental mental health problems, family conflicts, social support and personal resources were selected as predictors for the present analysis.

Overall, previous results of our COPSY study and the international literature led us to assume that there are at least two groups of different mental health trajectories. Thus, the objectives of this study are:

1. To explore whether distinct mental health, HRQoL and psychosomatic symptom trajectories in children and adolescents are identifiable over the course of the pandemic. We hypothesise that there is *at least one large group* of children and adolescents, who was resilient during the pandemic and showed either no impact or an initial decrease in mental health but recovered fast. Further, we hypothesise that there is *at least one group* characterised by decreasing mental health.
2. To investigate predictors of mental health groups differing in their trajectories of improvement, stability or deterioration. We assume that age, gender, low parental education and migration background as well as single parents households, financial or other pandemic burden, previous mental health problems, family conflicts, low social support, and low personal resources may be associated with an increased risk of belonging to the identified low mental health group(s).

## Methods

### Study design and sample

The German population-based longitudinal COPSY study assessed children, adolescents and their parents at five time points: At the start of the pandemic, when there was a partial lockdown in Germany: 05–06/2020 (T1), during the first pandemic winter with a full nationwide lockdown: 12/2020–01/2021 (T2), after the summer in the second year, when infection rates were low and restrictions loosened: 09–10/2021 (T3), at the end of the second pandemic winter, when there were still regulations of private gatherings: 02/2022 (T4), and in autumn of year three, when only minimal restrictions remained: 09–10/2022 (T5). In each survey wave, between *N* = 1586 (T1) and *N* = 1701 (T5) families participated [11, 23]. In this study, only the data of those *n* = 744 participants with complete data at all five measurement points were included who were aged 7 to 20 years and for whom parent-proxy report questionnaire data were available for all five measurement points. Respondents attending all five measurement points were older (1.3 years), were less likely to have a migration background and were more likely to have low parental education than respondents attending fewer measurements points. However, effect sizes were marginal. For single parenting, gender and occupation no statistically significant differences were observed. Regarding the mental health outcomes, a statistically significant difference was found for externalising mental health problems in three out of five measurements points, and for internalising mental health problems as well as for psychosomatic complaints in one out of five measurements points. Those participants attending all five measurements points displayed lower problems, however effect sizes again were only marginal. Families were invited via email to participate in the nationwide online survey which used quota sampling. This method helped to ensure that the sample reflected the sociodemographic characteristics of the German population. Detailed information on the study design and sample are published elsewhere [23, 24].

### Measures

The study included internationally established and validated instruments to assess the mental health of children and adolescents. HRQoL was measured via the parent-reported KIDSCREEN-10 Index [25]. Means were calculated with higher values indicating higher HRQoL. Mental health problems were assessed using the parent-reported Strengths and Difficulties Questionnaire (SDQ) [26], which provides a total difficulties’ score across 20 items and four subscales. Internalising (subscales emotional problems and peer problems) and externalising (subscales hyperactivity and conduct problems) scores were calculated, each ranging between 0 and 20, with higher scores indicating more severe problems. The frequencies of psychosomatic complaints like stomachaches, headaches, sleeping problems and irritability were measured via the parent-reported HBSC Symptom Checklist (HBSC-SCL) [27]. The mean item score was calculated ranging from 1 to 5, with higher values indicating more psychosomatic complaints. All HRQoL and mental health measures (KIDSCREEN-10 Index, SDQ, HBSC-SCL) are internationally validated and have good psychometric properties, which have been described in detail previously [12].

Familial resources were assessed using four items from the self-reported Family Climate Scale [28] and social support was measured using four items from the Social Support Scale [29]. Higher mean values indicated more pronounced resources.

For sociodemographics, age and gender of the children and adolescents as well as their parents were assessed. The parents also reported their marital status, education, living space, and migration background. Children, adolescents, and their parents were further asked about their perceived burden of the pandemic using single items.

### Statistical analyses

Mental health trajectories were explored using GMM to identify groups of children and adolescents, whose mental health changed in similar ways over the course of the pandemic. GMM is a person-centered approach, based on the assumption that the changes over the t = 5 measurement points in time can be adequately described if k distinct heterogeneous trajectory types underly [30–37]. Each trajectory represents an underlying latent class. For all members of a specific latent class, the same linear development over time can be assumed. More specific, the GMM assumes that the observed data are generated by a mixture of multiple Gaussian normal distributions, each representing a distinct latent class (c_j_; j = 1…k; unobserved subpopulations) [32, 34]. Different linear growth models are determined for each latent class. Within the GMM framework [37] the following assumptions were made: (1) Each person i belongs to one of k latent classes. (2) Each of the k classes is characterised by the specific linear function ${y}_{t|j}={\beta }_{0j}+{\beta }_{0j}\cdot {t}_{s}$. The GMM approach uses a restricted maximum-likelihood approach to determine (1) the probability of belonging to one of the corresponding classes 1...k for each person i (p$({c}_{j}\left|i\right))$, and (2) the most likely regression parameter b_0j_ and b_1j_ for each class k [30, 33]. The estimation was done by determining the optimal values b_0j_ and b_1j_ under the assumption of j classes (1 < j < m). Based on the χ^2^-fit-value and the number of model parameters to be estimated, the information-theoretical Bayesian information criterion (BIC) was determined. The number of classes j, for which the BIC shows the optimal combination of data fit and parsimony (i.e. minimum BIC value), indicate the most plausible class solution [35]. Additionally, the Lo–Mendell–Rubin adjusted Likelihood-Ratio (LMR-LR) test [31] indicated whether assuming j classes in contrast to j − 1 classes accounts for an incremental data fit: significant values indicated that the assumption of j classes is superior to the assumption of j − 1 classes.

Mplus was used to estimate the GMM [35]. To avoid convergence in local maxima indicating invalid global solutions, which may have resulted in skewed, non-normal data distributions [30], Mplus used random starting values. Furthermore, the Expectation-Maximization-Algorithm was used in the iterative estimation process.

Bivariate associations of the latent classes with categorial variables of T1 were analysed by χ^2^-based techniques including the estimation of association strength by the contingency coefficient (CC). Class associations with continuous indicators were analysed by one-way analysis of variance and the effect sizes measure η (small: η = 0.1, medium: η = 0.25, strong: η = 0.37) [36]. To quantify the respective change in each class between T1 and T5, a pairwise comparison of mean values was conducted using the effect size measure Cohen’s d, with 0.2 being interpreted as a small, 0.5 as a medium and 0.8 as a strong effect. In order to be able to ensure the results of the variance-analytical testing of class differences in covariates with regard to distribution problems (e.g. floor effects), the tests were additionally examined using the non-parametric Kruskal–Wallis test.

## Results

### Sociodemographics

The sociodemographic characteristics of the sample of *n* = 744 children and their parents are presented in Table 1. The age range of the children was between 7 and 20, with an initial mean of 12 years (SD = 3.3), the parents had a mean age of 44 years (SD = 7.1). There were slightly more boys reported upon than girls (51% vs. 49%). Most parents (59%) had a medium level of education, 14% had a migration background, 18% were single parents, 50% were full-time and 29% part-time employed.

**Table 1:** Sociodemographic characteristics of the COPSY sample

	Children and adolescents (T1)	Children and adolescents (T5)	Parents (T5)
	(*n* = 744)	(*n* = 744)	(*n* = 744)
Age (M [SD])	11.95 [3.28]	14.30 [3.32]	44.27 [7.13]
Gender (%)			
Male	51.14		48.8
Female	48.86		51.1
Migration background (%)			
No			86.02
Yes			13.98
Parental education (%)^1^			
Low			18.91
Medium			59.05
High			22.04
Single parenting (%)			
No			82.26
Yes			17.74
Occupational status (%)			
Full-time employed			50.40
Part-time employed			28.80
Self-employed			3.60
Other employment			2.00
Stay-at-home parent			8.30
Retiree/pensioner			3.00
On parental leave			1.70
Unemployed			2.20

*M* mean, *SD* standard deviation

^1^ Levels of education were operationalized based on the international ‘Comparative Analysis of Social Mobility in Industrial Nations’ (CASMIN) classification. This classification differentiates levels of education based on distinct combinations of academic and vocational qualifications. Based on these combinations, a categorization into parents with low (primary), medium (secondary), and high (tertiary) education was performed

### Trajectories of mental health in children and adolescents

Mental health trajectories were investigated for internalising and externalising mental health problems, psychosomatic symptoms, and HRQoL. Using GMM, four to five classes were found for the former three outcomes—with the best data fit considering the number of estimated model parameters used. For HRQoL, the GMM showed an optimal data fit for the one-class solution, suggesting that a homogeneous change model valid for all can be assumed. Table 2 shows the class characterisations of internalising MHP, externalising MHP, and psychosomatic symptoms by associations to sociodemographics, risk and resource factors.

**Table 2:** Associations between sociodemographics, risk and resource factors with the latent classes for internalising problems, externalising problems and psychosomatic symptoms

	Internalising mental health problems					Externalising mental health problems				Psychosomatic symptoms			
	Chi^2^		df	p	CC	Chi^2^	df	p	CC	Chi^2^	df	p	CC
Gender	1.53		3	0.675	0.045	11.10	4	0.025	0.121	3.58	4	0.466	0.069
Migration background	2.11		3	0.551	0.053	0.76	4	0.944	0.032	8.06	4	0.089	0.104
Parental education	3.60		6	0.731	0.070	3.08	8	0.930	0.065	8.15	8	0.419	0.105
Single parenting	7.19		3	0.066	0.098	4.07	4	0.396	0.074	7.38	4	0.117	0.099
COVID in family	0.55		3	0.907	0.027	3.71	4	0.447	0.070	6.55	4	0.161	0.093
COVID infected child	1.31		3	0.728	0.042	4.64	4	0.326	0.079	6.19	4	0.191	0.090

	Internalising mental health problems					Externalising mental health problems					Psychosomatic symptoms				
	F; df = 3,740	p	η	H df = 3	p	F; df = 3,740	p	η	H df = 4	p	F; df = 3,740	p	η	H df = 4	p
Age	1.47	0.222	0.077	4.09	0.252	12.97	< 0.001	0.256	48.93	< 0.001	1.41	0.231	0.057	5.53	0.237
Living space	2.51	0.057	0.100	8.37	0.039	2.28	0.059	0.110	9.46	0.051	2.00	0.093	0.104	8.03	0.091
HRQoL	63.19	< 0.001	0.204	162.26	< 0.001	42.70	< 0.001	0.188	155.52	< 0.001	37.63	< 0.001	0.169	132.66	< 0.001
Parental burden	9.59	< 0.001	0.193	24.16	< 0.001	13.21	< 0.001	0.290	59.31	< 0.001	10.18	< 0.001	0.228	36.19	< 0.001
Burden of the child	14.84	< 0.001	0.289	37.53	< 0.001	5.98	< 0.001	0.224	24.27	< 0.001	11.09	< 0.001	0.298	41.20	< 0.001
Personal resources	36.24	< 0.001	0.439	74.39	< 0.001	10.40	< 0.001	0.290	30.83	< 0.001	21.03	< 0.001	0.395	56.53	< 0.001
Family climate	14.12	< 0.001	0.292	35.13	< 0.001	8.68	< 0.001	0.267	32.72	< 0.001	8.68	< 0.001	0.266	32.84	< 0.001
Social support	5.95	< 0.001	0.194	15.51	< 0.001	5.32	< 0.001	0.211	17.69	< 0.001	5.39	< 0.001	0.213	17.44	0.002

η (eta): 0.1– small, 0.24– middle and 0.37– large effect size; H = Kruskal–Wallis H

*CC * Concordance coefficient

### Internalising mental health problems

**Fig. 1:**
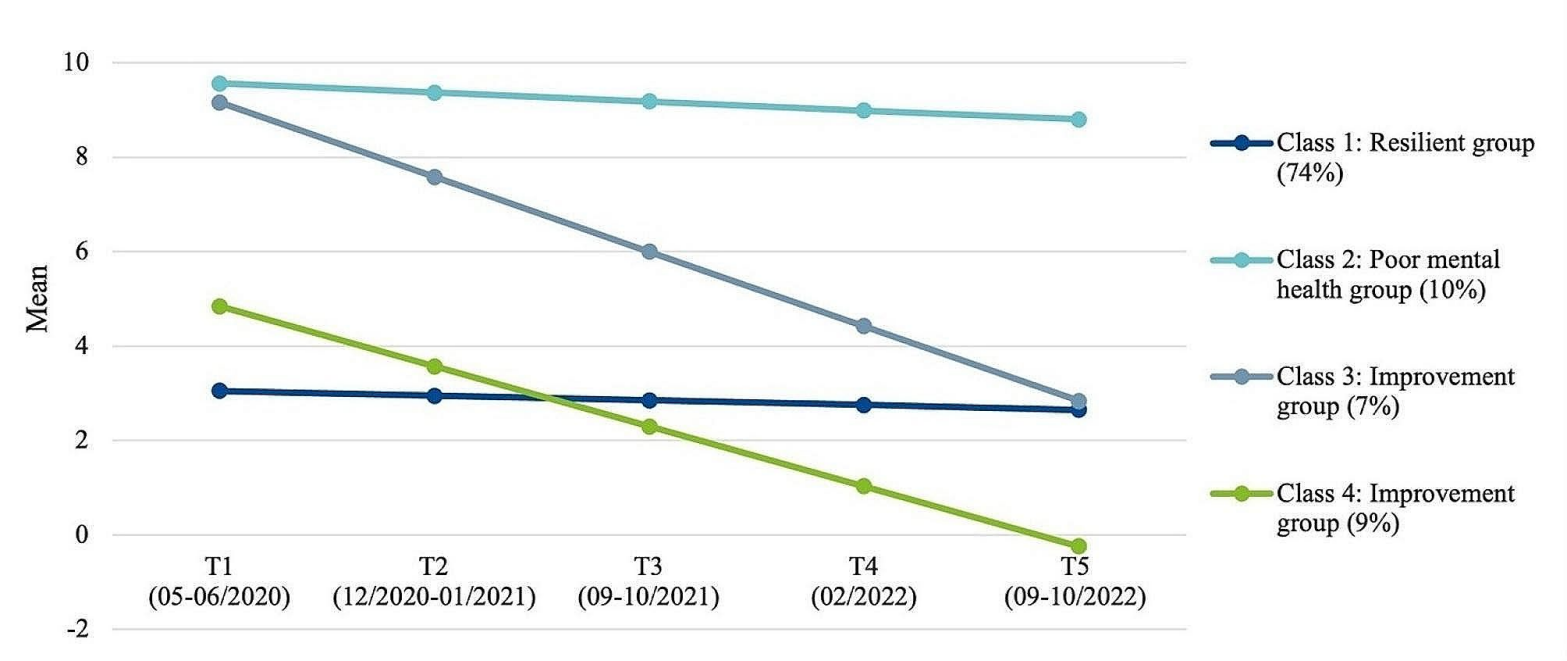
Trajectories of internalising mental health problems in children and adolescents during the COVID-19 pandemic

We found that four distinct mental health trajectory groups best described the data for internalising MHP (BIC: 17230.58, LMR-LR: 79.46, *p* =.002). The four groups are illustrated in Fig. 1 and described in Table 3. The largest first class (*resilient group*, 74%) remained almost stable across the three pandemic years with very slight improvements at a low internalising symptom level (M_T1_ = 3.1 to M_T5_ = 2.7, Cohen’s d = − 0.24). Participants belonging to this group showed the highest HRQoL, the lowest pandemic burden, high personal resources, the best family climate and best social support across all four classes. The second class (*poor mental health group*) with 10% of the participants started at a high symptom level at the beginning of the pandemic and remained at a high symptom level, improving only slightly (M_T1_ = 9.6 to M_T5_ = 8.8, Cohen’s d = − 0.45). This group included participants with the lowest HRQoL, feeling most burdened by the pandemic and having the lowest personal resources and worst family climate compared to the other classes. The third (7%) and fourth class (9%) showed substantial improvements in internalising MHP (*improvement groups*) across the pandemic.

**Table 3:** Results of the covariate analysis for internalising problems, externalising problems and psychosomatic symptoms

	Age (at T1)	HRQoL	Parental burden	Child burden	Personal resources	Family climate	Social support
	M(SD)	M (SD)	M (SD)	M (SD)	M (SD)	M (SD)	M (SD)
Internalising MHP							
Class 1	12.06 (3.30)	43.96 (9.06)	3.15 (0.91)	2.85 (0.99)	72.05 (15.06)	77.75 (17.56)	79.23 (16.84)
Class 2	11.27 (3.00)	31.79 (7.31)	3.68 (0.83)	3.87 (0.94)	47.89 (20.50)	59.65 (21.09)	70.89 (18.28)
Class 3	12.04 (3.35)	33.21 (6.10)	3.50 (0.79)	3.43 (0.86)	51.78 (19.07)	67.50 (20.92)	70.83 (18.59)
Class 4	11.64 (3.26)	38.62 (8.18)	3.34 (0.96)	3.15 (1.09)	67.18 (20.31)	69.87 (19.16)	71.47 (21.04)
Externalising MHP							
Class 1	12.46 (3.32)	44.49 (9.01)	3.07 (0.90)	2.87 (1.01)	71.30 (15.97)	78.10 (17.84)	79.50 (18.85)
Class 2	10.48 (3.24)	36.40 (7.56)	3.57 (0.79)	3.17 (1.21)	60.44 (17.41)	66.39 (20.47)	72.08 (19.68)
Class 3	11.89 (2.85)	38.48 (9.36)	3.32 (0.91)	3.20 (1.02)	63.57 (20.18)	68.12 (20.36)	73.37 (16.80)
Class 4	10.11 (3.05)	33.12 (7.06)	3.90 (0.78)	3.69 (0.84)	52.82 (23.99)	64.74 (21.12)	66.59 (22.36)
Class 5	10.69 (3.03)	32.17 (7.15)	3.54 (0.88)	3.67 (0.52)	65.56 (26.13)	65.28 (11.08)	77.08 (20.03)
Psychosomatic symptoms							
Class 1	11.94 (3.32)	43.32 (9.28)	3.15 (0.90)	2.87 (1.0)	71.17 (15.93)	77.39 (18.09)	78.75 (16.98)
Class 2	12.26 (3.24)	32.26 (6.15)	3.69 (0.76)	3.74 (0.79)	55.81 (19.95)	62.21 (19.62)	66.42 (19.05)
Class 3	11.84 (2.85)	41.87 (6.77)	3.23 (0.99)	2.93 (0.98)	71.19 (15.51)	69.94 (20.71)	79.46 (14.82)
Class 4	11.03 (3.05)	31.04 (8.15)	3.84 (0.85)	3.87 (0.99)	43.56 (20.76)	68.89 (24.69)	74.58 (23.68)
Class 5	13.42 (3.03)	34.67 (8.58)	3.67 (1.1)	3.40 (1.3)	47.33 (25.43)	62.50 (12.58)	70.63 (21.86)

### Externalising mental health problems

**Fig. 2:**
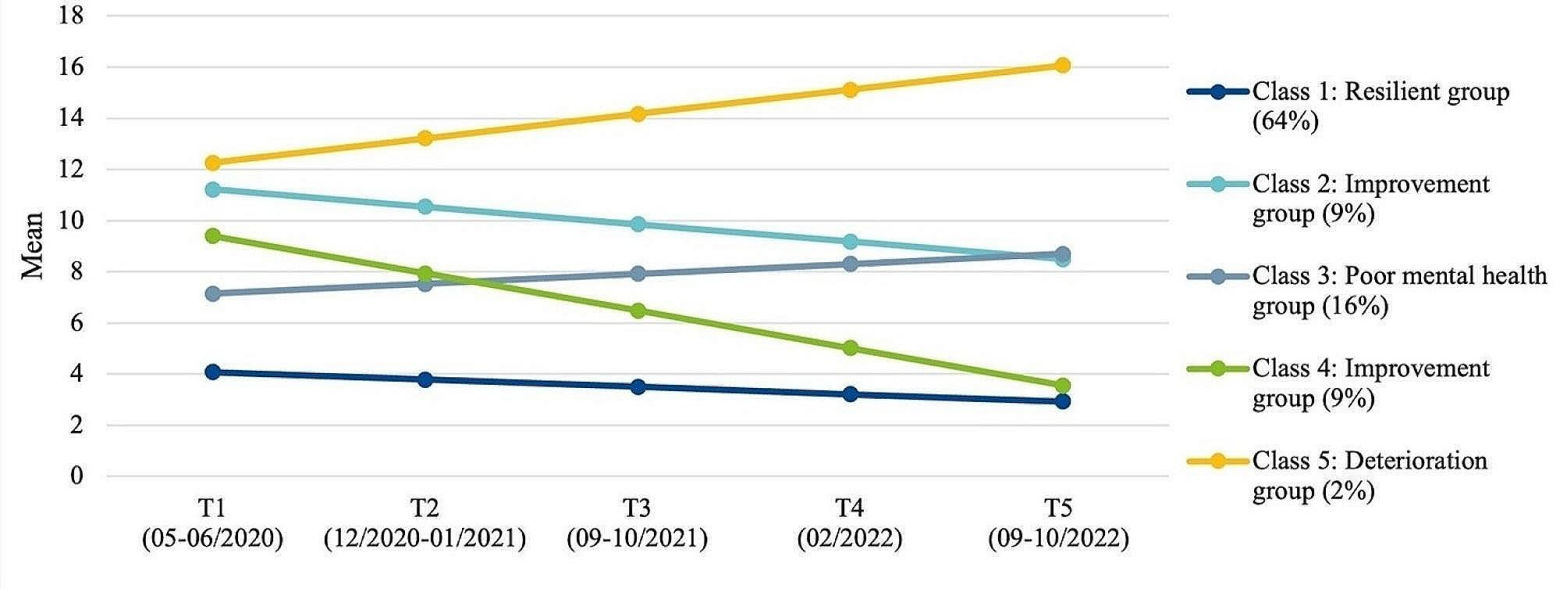
Trajectories of externalising mental health problems in children and adolescents during the COVID-19 pandemic

For externalising MHP, the best model fit of GMM analyses was found for a five trajectory groups solution (BIC: 16990.61, LMR-LR: 27.34, *p* =.184) as displayed in Fig. 2 and Table 3. Again, the largest first class (*resilient group*, 64%) remained almost stable with slight improvements at a low externalising symptom level (M_T1_ = 4.1 to M_T5_ = 2.9, Cohen’s d = − 0.32) across the three pandemic years. Youths in that group tended to be more likely 12 than 10 years old, reported the highest HRQoL, the lowest child and parental burden, and the best personal resources, family climate and social support compared to the other four classes. The second largest class (*poor mental health group*, 16%) showed increasing externalising symptoms from M_T1_ = 7.2 to M_T5_ = 8.7 (Cohen’s d = 0.43), being rather 12 than 10 years old and having medium HRQoL and a low pandemic burden. Personal and familial resources were medium in that group. Further, two *improvement groups* (class 2 and 4, both 9%) showed a significant decrease (Cohen’s d = − 0.76) in externalising symptoms across the five survey waves. And finally, the smallest group (*deterioration group*, 2%) starting at a high symptom level showed a substantial symptom increase across the pandemic (Cohen’s d = 1.06).

### Psychosomatic symptoms

**Fig. 3:**
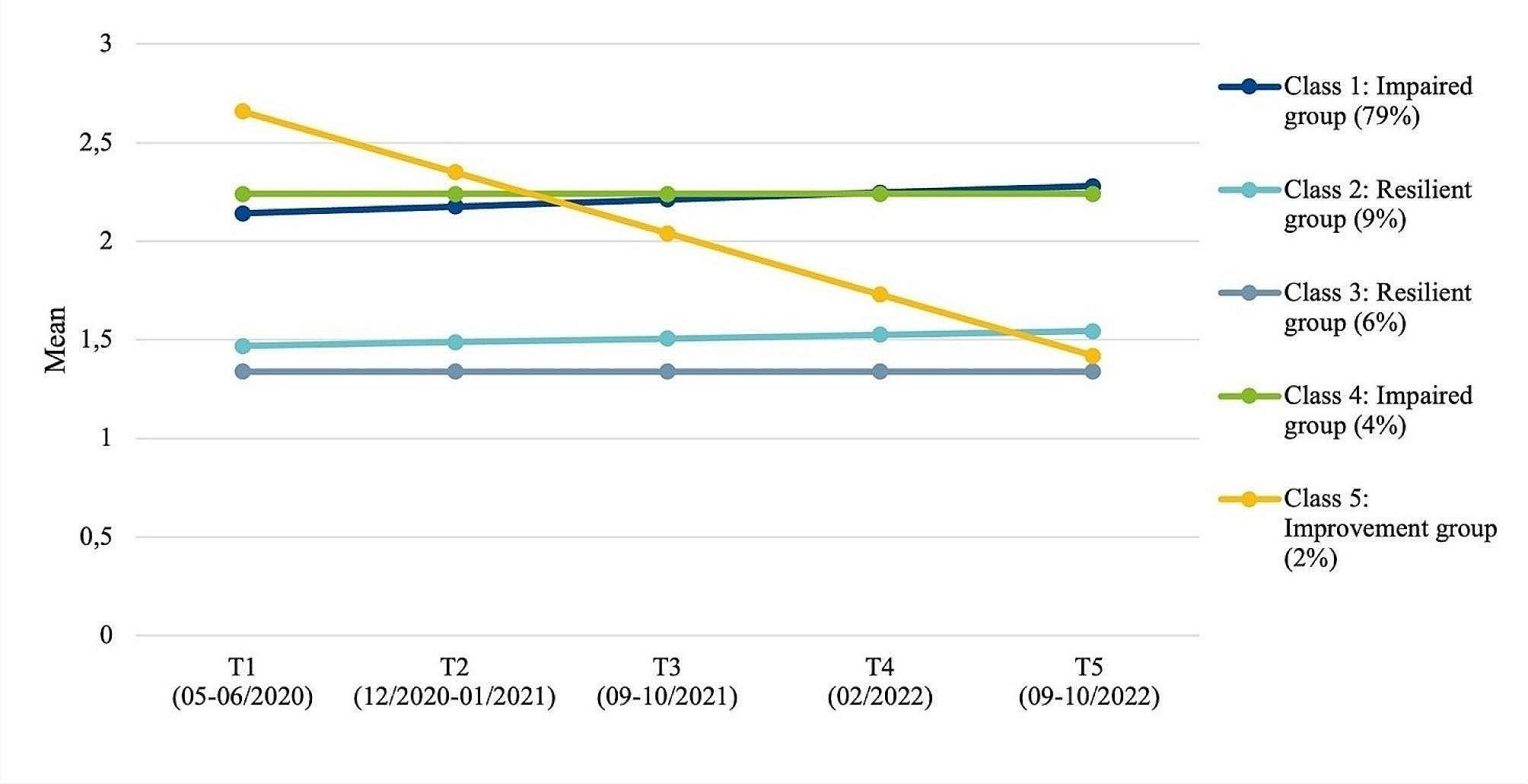
Trajectories of psychosomatic symptoms in children and adolescents during the COVID-19 pandemic

With the GMM analyses a five psychosomatic symptom trajectory groups solution exhibited the most appropriate model fit (BIC: 3293.84, LMR-LR: 163.56, *p* =.007) (see Fig. 3; Table 3). The largest first class (*impaired group*, 79%) showed slightly worsening moderate symptoms (M_T1_ = 2.1 to M_T5_ = 2.3, Cohen’s d = 0.28). This largest group can be characterised by a high HRQoL, the least child and parental burden, very good personal resources, the best family climate and high social support across the five classes. Interestingly there is a very small group (4%), which has a very similar but more stable trajectory (Cohen’s d = 0.00) with the highest perceived child and parental burden and lowest personal resources of all those classes, yet a medium family climate. Parallel to those trajectories, two *resilient groups* (6 and 9%) showed stable trajectories (Cohen’s d = 0.15/0.00) with no to very few psychosomatic symptoms. Finally, there is one class (*improvement group*, 2%), which initially had moderate psychosomatic symptoms, but show a steep decline of psychosomatic symptoms across the pandemic (Cohen’s d = − 2.5). This group had a rather low initial HRQoL, was burdened the most initially and shows a medium child and parental burden, quite low initial personal resources and a bad family climate.

Further findings of the covariate analyses indicated that there were few and rather weak associations between the class categorization of the three scales and most sociodemographic or COVID-related factors (see Table 2). Significant associations between the classes of each construct were found in the covariate analyses as displayed in Table 2 for parental burden (η = 0.193 to 0.290, *p* <.001), burden of the child (η = 0.224 to 0.298, *p* <.001), personal resources (η = 0.290 to 0.439, *p* <.001), family climate (η = 0.266 to 0.292, *p* <.001) and social support (η = 0.194 to 0.213, *p* <.001).

Class memberships proved to be highly correlated: *χ*^2^_df = 12_ = 389.29 (*p* <.001) and CC = 0.584 for internalising and externalising MHP classes, *χ*^*2*^_df = 12_ = 306.782 (*p* <.001) and CC = 0.540 for psychosomatic symptoms and internalising MHP, and *χ*^*2*^_df = 12_ = 275.51 (*p* <.001) for psychosomatic symptoms and externalising MHP. Members of internalising MHP class 1 (resilient group) and 3 (improvement group) were most likely to belong to class 1 (resilient group) for the externalising MHP (80.0% and 46.0%). For the members of internalising MHP class 2 (poor mental health group) and 4 (improvement group), however, externalising MHP class 3 (poor mental health group) was most probable (39.7% and 58.2%). Nine of the 13 members (69.2%) of externalising MHP class 5 (deterioration group) belonged to internalising MHP class 4 (improvement group). Psychosomatic symptom class 1 (impaired group) mostly included both resilient members in the internalising (class 1; 85.9%) and externalising (class 1; 75.0%) MHP. Members of psychosomatic symptom class 2 (resilient group) or 5 (improvement group) were most likely to be in class 2 (poor mental health group) for internalising (42.6%, 41.7%) and class 3 (poor mental health group) for externalising MHP (32.4%, 61.4%). Members of psychosomatic symptoms class 3 (resilient group) were also most likely to be resilient in the area of internalising MHP (class 1; 54.5%) and to be in class 3 (poor mental health group) for externalising MHP (61.4%). Class 4 (impairment group) with regard to psychosomatic symptoms mainly includes members of internalising MHP class 3 (improvement group) (37.5%) and externalising MHP class 2 (improvement group) (40.6%). The contingency tables of the complete data distributions are attached in the Online Appendix A.

## Discussion

This is one of the first studies using in-depth general mixture modeling of longitudinal mental health data over almost three years of the COVID-19 pandemic in children and adolescents. Therewith our COPSY study goes beyond reporting average statistics of mental health by investigating trajectories of children and adolescents. We identified four (internalising MHP) to five (externalising MHP and psychosomatic symptoms) distinct latent mental health trajectory classes. For HRQoL a single class solution was found to be appropriate. Thus, for HRQoL GMM analysis does not provide any further indications of informative differentiations of change processes that provide more detailed information on previously published COPSY findings. This was surprising to us and should be investigated in more detail in future studies. HRQoL can be defined as a global multidimensional construct which describes physical, social and psychological aspects of well-being and functioning [38], so it could be possible that it is too complex and global that different HRQoL trajectory groups could not be found.

The present study found that almost three quarters of children and adolescents had consistently low internalising (64%) and externalising symptom levels (74%). Children belonging to this *resilient group* had good mental health and stayed mentally stable during the pandemic, confirming our *first hypothesis* that (at least) one group showed no pandemic impact. According to a prepandemic systematic review [13], a one large class solution with no or only minor symptoms is common when applying LCA/GMM to population-based mental health data. Our study confirms those findings for internalising and externalising MHP in times of health crises. A recent review on adult mental health studies using GMM [16] specified that, similar to a pre-pandemic meta-analysis [15], during the pandemic the pooled prevalence of the *resilient* groups was 66% in adults, which is between the 64% and 74% of resilient adolescents of the present study.

However, this pattern could not be found for psychosomatic symptoms. The majority (79%) of youths were allocated to the *impaired group* reporting moderate psychosomatic symptoms (once to four times per week), suggesting that psychosomatic reactions have been quite common since the start of the pandemic. This is in line with our previous COPSY publications [11]. The most recent European HBSC study details that psychosomatic symptoms have been particularly frequent in 15-year-old girls with 50% of them reporting psychosomatic symptoms more than once a week during the pandemic [39]. Another cross-national European study covering pre-pandemic data of children found that 46% had at least one frequent psychosomatic symptom during the past six months [40], which is similar to a review on adolescents and adults, which describes that 40 to 49% complained about at least one medically unexplained/psychosomatic symptom [41]. To summarise, findings of the present study indicate a strong increase in psychosomatic symptoms compared to the outlined large-scale studies and reviews. This might be explained by the fact that the COVID-19 infection itself led to psychosomatic complaints, that possible post-infection symptoms persisted or that children and adolescents psychosomatically expressed their worries or other emotions. However, evidence regarding these associations is limited and further studies are needed.

Moreover, the present study found three *improvement groups* for internalising and externalising MHP as well as psychosomatic symptoms. Thus, 2 to 18% of the initially moderately to severely impaired children and adolescents recovered over the pandemic. This also seems similar to the pooled prevalence of 13% allocated in *recovery* groups found in a review of adult studies during the pandemic using GMM [16]. Our improvement groups found could be interpreted as meaning that the interests and needs of children and adolescents were heard and met after neglecting their mental health at the beginning of the pandemic, e.g., by adopting strict lockdown measures, so living conditions for these children may have improved.

Nevertheless, this study also revealed that a tenth of all children and adolescents (10%) have suffered from internalising MHP throughout the pandemic. This is very similar to a *chronic* adult mental health trajectory group (11%) found in pre- and pandemic adult studies using GMM according to reviews [15, 16]. In terms of externalising MHP, almost every fifth youth (18%) has had persistent or even increasing hyperactivity and conduct problems since the start of the pandemic. Those groups were called the *poor mental health groups* and the *deterioration group*, confirming the second part of our *first hypothesis* that there is at least one group that has suffered throughout the pandemic and is still burdened in the third year. This is in line with our previous research indicating that mental health problems increased during the pandemic and stabilised on an impaired level [11]. These groups of children and adolescents need special attention in the context of prevention, diagnostics and mental health care.

The current study holds the advantage that it utilised GMM for an in-depth exploration of a longitudinal class characterisation of the sample. Many longitudinal pandemic paediatric studies using average statistics may oversimplify the complexity of intra- and interindividual variability [42]. To our knowledge there are only few paediatric mental health studies, which used latent class modeling during pandemic times [43–46], most of which were cross-sectional studies. To our knowledge there is only one longitudinal adolescent study [47], which applied LCA in a similar manner as our COPSY study. Wang et al. [47] assessed mental health in a large Chinese adolescent’s sample aged 16–25 years in 2020. They found two subgroups—called resistance vs. dysfunction—of adolescents based on their depressive and anxiety symptoms. In the whole sample they identified a subthreshold symptom increase in year 1, but when stratifying the sample in the two subgroups from February to June 2020, the larger resistance group (93%) had low depressive symptoms, which slightly increased but remained subthreshold, while the smaller dysfunction group (7%) had high depressive symptoms, which improved during the summer of 2020, but remained above the clinical cut-off score. The poor mental health groups found in our and the mentioned study may be related to risk factors like pre-existing mental or physical health problems, age, gender, a low socio-economic status, infection risk, lockdowns, low self-regulating abilities, low parental mental health or parenting quality, low family functioning, social support, isolation, loneliness, health-related worries or inconsistent routines (for a summary of risk factors, see [6]).

While in previous studies, some of the above-mentioned risk factors were found, this study surprisingly found that class membership was neither associated with any sociodemographic factors nor a COVID infection, which was contrary to our hypothesis 2—except for a small gender and age association for some groups of youths allocated in externalising MHP classes.

In line with *hypothesis 2*, we found that class membership was significantly associated with HRQoL, initial parental and child pandemic burden, personal resources, family climate and social support. Children and adolescents feeling burdened or with parents feeling burdened at the start of the pandemic felt worse during the pandemic than their peers. Youths with good personal resources, a good family climate and social support at the start of the pandemic were more frequently assigned to the resilient or improvement groups. This is in line with previous research on risk and protective factors, indicating that parental burden and psychopathology are associated with an increased risk of impaired mental health in children and adolescents during the pandemic, whereas personal, familial, and social resources support children and adolescents in staying mentally healthy [12, 17].

The additional analyses regarding the associations between the class memberships of internalising, externalising and psychosomatic symptoms revealed high correlations between all classes of the constructs, indicating a strong content validity. The analyses found the largest association of the class members (80%) between the resilient internalising and resilient externalising classes and the largest association of class members of the psychosomatic impaired and both resilient internalising and externalising classes (86% and 75%). This could be due to the size of those classes which make up the majority of the youths analysed. The other associations are mixed and seem to be partly arbitrary, i.e. there were improved children in the same or the opposite classes of the other constructs with 31–61% overlap.

Findings of the present study, together with evidence from previous studies, suggest that more detailed analysis of mental health data is highly informative and worthwhile, as these methods help to expand our knowledge of interrelated factors in mental health and subgroups of mental health status at a given time point. For longitudinal data, trajectory modeling techniques like LCA and GMM or more individual-centered statistical approaches such as cluster analysis and sequence analysis produce important information on the evolution and progression of mental health subgroups over time, which is particularly needed in times of crisis like the COVID-19 pandemic. We would like to encourage researchers in the field to use these methods more frequently longitudinally to inform children’s mental health care professionals and policymakers.

The strengths of this study are: (1) a large population-based longitudinal sample of children and adolescents surveyed over a period of almost three years during the pandemic; (2) the use of internationally established and validated questionnaires; and (3) the application of advanced statistical methods. Limitations are: (1) that the results may not be generalizable to other populations or countries as the study was conducted in the context of the specific pandemic situation in Germany, which also differs from other countries worldwide in terms of wealth and aspects of healthcare (provision); (2) the SDQ and HBSC-SCL were used in this study, which, due to the item formulations and recorded content areas, exhibit floor effects in non-clinical, population-based samples. The characteristics of the identified classes must therefore be interpreted with regard to the rather demanding formulation (“minor problems” means minor problems understood in a clinical sense). It must be assumed that the dominance of the “resilient group” as the largest class is therefore also due to the low variability of the distribution of characteristics; (3) GMM is a data-driven, structure-identifying exploratory approach, for which replicative studies would be desirable for validation due to the lack of a priori hypothesis. Especially small classes could be statistical artifacts lacking reproducibility [48], thus we carefully selected the model using the fit indices described and conducted covariate analyses to substantiate their clinical meaningfulness. Although some of the identified classes are small, the identified class sizes are still above the critical limit of < 1% described in the literature. In addition to considering the class-specific fit indicators, the LMR-LR test was used to explicitly examine the need to consider the small classes in order to better and adequately describe the data information [49]; (4) there may be some content overlap between the investigated outcomes/scales (e.g. a few HRQoL items may be similar to SDQ internalising items), which may affect the discriminative interpretation of the results; (5) statistical power issues cannot be resolved in general terms, as the size of the classes to be identified is a decisive factor in this respect [49, 50]. For this reason, we chose an analysis approach that was as economic as possible, in that only those study participants who took part in all five measurement points were included in the data analysis. In addition, only linear trajectories were modeled, as estimating non-linear trajectories would have led to a disproportionate increase in the number of estimated parameters. The linearity assumption implies that only general stability and continuous improvement and deterioration during the pandemic could be identified; and (6) only those study participants who took part in all five measurement points were included in the data analysis. It cannot be ruled out that those excluded were not missing completely at random [51] and thus may bias the study results.

## Conclusions

The mental health of children and adolescents has deteriorated during the COVID-19 pandemic, yet evidence on developmental trajectories based on longitudinal studies was limited. The population-based longitudinal COPSY study examined mental health trajectories and potential predictors associated with improvement, stability or deterioration. Most children and adolescents had good mental health and were resilient during the three pandemic years. However, one tenth to one fifth of the children had consistently high or deteriorating mental health problems, especially if they had low personal, familial and social resources, which call for special attention in schools and mental health care. Our findings provide mental health professionals and policymakers with evidence that can be used to support youth mental health in times of crises.

### Electronic supplementary material

Below is the link to the electronic supplementary material.

Supplementary Material 1

## Data Availability

The data that support the findings of this study are available from the corresponding author upon reasonable request.

## Abbreviations

BIC: Bayesian information criterion
CC: Contingency coefficient
COVID-19: Coronavirus disease 2019
COPSY: COvid-19 and PSYchological Health
GMM: Generalized mixture modeling
HBSC-SCL: Health Behaviour in School-aged Children Symptom Checklist
HRQoL: Health-Related Quality of Life
LCA: Latent class analyses
LMR-LR: Lo-Mendell-Rubin adjusted Likelihood-Ratio
MHP: Mental health problems
SDQ: Strengths and Difficulties Questionnaire

## References

[1] Kauhanen L, Wan Mohd Yunus WMA, Lempinen L, Peltonen K, Gyllenberg D, Mishina K, Gilbert S, Bastola K, Brown JS, Sourander A. A systematic review of the mental health changes of children and young people before and during the COVID-19 pandemic. Eur Child Adolesc Psychia-try. 2023;32:995–1013. 10.1007/s00787-022-02060-0. 10.1007/s00787-022-02060-0 PMC9373888 35962147

[2] Ma L, Mazidi M, Li K, Li Y, Chen S, Kirwan R, Zhou H, Yan N, Rahman A, Wang W. Prevalence of mental health problems among children and adolescents during the COVID-19 pandemic: a systematic review and meta-analysis. J Affect Disord. 2021;293:78–89. 10.1016/j.jad.2021.06.021. 34174475 10.1016/j.jad.2021.06.021 PMC9711885

[3] Racine N, McArthur BA, Cooke JE, Eirich R, Zhu J, Madigan S. Global prevalence of depressive and anxiety symptoms in children and adolescents during COVID-19: a meta-analysis. JAMA Pediatr. 2021;175:1142–50. 10.1001/jamapediatrics.2021.2482 10.1001/jamapediatrics.2021.2482 PMC8353576 34369987

[4] Samji H, Wu J, Ladak A, Vossen C, Stewart E, Dove N, Long D, Snell G. Mental health impacts of the COVID-19 pandemic on children and youth—a systematic review. Child Adolesc Psychiatry Ment Health. 2022;27:173–89. 10.1111/camh.12501 10.1111/camh.12501 PMC8653204 34455683

[5] Schlack R, Neuperd L, Junker S, Eicher S, Hölling H, Thom J, Ravens-Sieberer U, Beyer A-K. Veränderungen Der Psychischen Gesundheit in Der Kinder-Und Jugendbevölkerung in Deutschland während Der COVID-19-Pandemie–Ergebnisse eines Rapid Reviews. J Health Monit. 2023;S1:1–74

[6] Wolf K, Schmitz J. Scoping review: longitudinal effects of the COVID-19 pandemic on child and adolescent mental health. Eur Child Adolesc Psychiatry. 2023. 10.1007/s00787-023-02206-8. 37081139 10.1007/s00787-023-02206-8 PMC10119016

[7] Orban E, Li LY, Gilbert M, Napp A-K, Kaman A, Topf S, Böcker M, Devine J, Reiss F, Wendel F, Jung-Sievers C, Ernst VS, Franze M, Möhler E, Breitinger E, Bender S, Ravens-Sieberer U. Mental health and quality of life in children and adolescents during the COVID-19 pandemic: a systematic review of longitudinal studies. Front Public Health. 2024;11:1275917. 38259801 10.3389/fpubh.2023.1275917 PMC10800626

[8] Fischer K, Tieskens JM, Luijten MAJ, Zijlmans J, van Oers HA, de Groot R, van der Doelen D, van Ewijk H, Klip H, van der Lans RM, De Meyer R, van der Mheen M, van Muilekom MM, Hyun Ruisch I, Teela L, van den Berg G, Bruining H, van der Rijken R, Buitelaar J, Hoekstra PJ, Lindauer R, Oostrom KJ, Staal W, Vermeiren R, Cornet R, Haverman L, Bartels M, Polderman TJC, Popma A. Internalizing problems before and during the COVID-19 pandemic in independent samples of Dutch children and adolescents with and without pre-existing mental health problems. Eur Child Adolesc Psychiatry. 2022. 10.1007/s00787-022-01991-y 10.1007/s00787-022-01991-y PMC9133820 35616715

[9] Theuring S, van Loon W, Hommes F, Bethke N, Mall MA, Kurth T, Seybold J, Mockenhaupt FP. Psychosocial wellbeing of Schoolchildren during the COVID-19 pandemic in Berlin, Germany, June 2020 to March 2021. Int J Environ Res Public Health. 2022;19:10103. 10.3390/ijerph191610103 36011738 10.3390/ijerph191610103 PMC9407732

[10] Felfe C, Saurer J, Schneider P, Vornberger J, Erhart M, Kaman A, Ravens-Sieberer U. The youth mental health crisis: quasi-experimental evidence on the role of school closures. Sci Adv. 2023;9:eadh4030. 10.1126/sciadv.adh4030. 37595042 10.1126/sciadv.adh4030 PMC10438447

[11] Ravens-Sieberer U, Devine J, Napp A-K, Kaman A, Saftig L, Gilbert M, Reiß F, Löffler C, Simon AM, Hurrelmann K, Walper S, Schlack R, Hölling H, Wieler LH, Erhart M. Three years into the pandemic: results of the longitudinal German COPSY study on youth mental health and health-related quality of life. Front Public Health. 2023;11:1129073. 10.3389/fpubh.2023.1129073 37397777 10.3389/fpubh.2023.1129073 PMC10307958

[12] Ravens-Sieberer U, Kaman A, Erhart M, Otto C, Devine J, Löffler C, Hurrelmann K, Bullinger M, Barkmann C, Siegel NA, Simon AM, Wieler LH, Schlack R, Hölling H. Quality of life and mental health in children and adolescents during the first year of the COVID-19 pandemic: results of a two-wave nationwide population-based study. Eur Child Adolesc Psychiatry. 2021;32:575–88. 10.1007/s00787-021-01889-1. 34636964 10.1007/s00787-021-01889-1 PMC8506100

[13] Petersen KJ, Qualter P, Humphrey N. The application of latent class analysis for investigating population child mental health: a systematic review. Front Psychol. 2019;10:1214. 10.3389/fpsyg.2019.01214 31191405 10.3389/fpsyg.2019.01214 PMC6548989

[14] Wang D, Zhao J, Ross B, Ma Z, Zhang J, Fan F, Liu X. Longitudinal trajectories of de-pression and anxiety among adolescents during COVID-19 lockdown in China. J Affect Disord. 2022;299:628–35. 10.1016/j.jad.2021.12.086. 34952127 10.1016/j.jad.2021.12.086 PMC8691948

[15] Galatzer-Levy IR, Huang SH, Bonanno GA. Trajectories of resilience and dysfunction following potential trauma: a review and statistical evaluation. Clin Psychol Rev. 2018;63:41–55. 29902711 10.1016/j.cpr.2018.05.008

[16] Schäfer SK, Kunzler AM, Kalisch R, Tuscher O, Lieb K. Trajectories of resilience and mental distress to global major disruptions. Trends Cogn Sci. 2022;26:1171–89. 36302711 10.1016/j.tics.2022.09.017 PMC9595401

[17] Güzelsoy N, Ravens-Sieberer U, Westenhofer J, Devine J, Erhart M, Holling H, Kaman A. Risks and resources for depressive symptoms and anxiety in children and adolescents during the COVID-19 pandemic—results of the Longitudinal COPSY Study. Front Psychiatry. 2022;13:901783. 10.3389/fpsyt.2022.901783 35873222 10.3389/fpsyt.2022.901783 PMC9301280

[18] Zhang Q, Zhou Y, Ho SMY. Active and avoidant coping profiles in children and their relationship with anxiety and depression during the COVID-19 pandemic. Sci Rep. 2022;12:13430. 10.1038/s41598-022-15793-4. 35927558 10.1038/s41598-022-15793-4 PMC9352659

[19] Martinsone B, Stokenberga I, Damberga I, Supe I, Simoes C, Lebre P, Canha L, Santos M, Santos AC, Fonseca AM, Santos D, Gaspar de Matos M, Conte E, Agliati A, Cavioni V, Gandellini S, Grazzani I, Ornaghi V, Camilleri L. Adolescent social emotional skills, resilience and behavioral problems during the COVID-19 pandemic: a longitudinal study in three European countries. Front Psychiatry. 2022;13:942692. 10.3389/fpsyt.2022.942692 35978848 10.3389/fpsyt.2022.942692 PMC9376252

[20] Nikolaidis A, DeRosa J, Kass M, Droney I, Alexander L, Di Martino A, Bromet E, Merikangas K, Milham MP, Paksarian D. Heterogeneity in COVID-19 pandemic-induced lifestyle stressors predicts future mental health in adults and children in the US and UK. J Psychiatr Res. 2022;147:291–300. 10.1016/j.jpsychires.2021.12.058. 35123338 10.1016/j.jpsychires.2021.12.058 PMC8720815

[21] Bruining H, Bartels M, Polderman TJ, Popma A. COVID-19 and child and adolescent psychiatry: an unexpected blessing for part of our population? Eur Child Adolesc Psychiatry. 2021;30:1139–40. 10.1007/s00787-020-01578-5. 32623697 10.1007/s00787-020-01578-5 PMC7335225

[22] Cost KT, Crosbie J, Anagnostou E, Birken CS, Charach A, Monga S, Kelley E, Nicolson R, Maguire JL, Burton CL. Mostly worse, occasionally better: impact of COVID-19 pandemic on the mental health of Canadian children and adolescents. Eur Child Adolesc Psychiatry. 2022. 10.1007/s00787-021-01744-3. 10.1007/s00787-021-01744-3 PMC7909377 33638005

[23] Ravens-Sieberer U, Erhart M, Devine J, Gilbert M, Reiss F, Barkmann C, Siegel NA, Simon AM, Hurrelmann K, Schlack R, Hölling H, Wieler LH, Kaman A. Child and adolescent mental health during the COVID-19 pandemic: results of the three-wave longitudinal COPSY study. J Child Adolesc Ment Health. 2022;71:570–8. 10.1016/j.jadohealth.2022.06.022. 10.1016/j.jadohealth.2022.06.022 PMC9386895 35989235

[24] Ravens-Sieberer U, Kaman A, Erhart M, Devine J, Schlack R, Otto C. Impact of the COVID-19 pandemic on quality of life and mental health in children and adolescents in Germany. Eur Child Adolesc Psychiatry. 2022;31:879–89. 10.1007/s00787-021-01726-5. 33492480 10.1007/s00787-021-01726-5 PMC7829493

[25] Ravens-Sieberer U, KIDSCREEN Group Europe. The Kidscreen questionnaires: quality of life questionnaires for children and adolescents; handbook. Lengerich: Pabst Science Publ.; 2006.

[26] Goodman R. The strengths and difficulties Questionnaire: a research note. J Child Psy-chol Psychiatry. 1997;38:581–6. 10.1111/j.1469-7610.1997.tb01545.x. 10.1111/j.1469-7610.1997.tb01545.x 9255702

[27] Haugland S, Wold B. Subjective health complaints in adolescence—reliability and validity of survey methods. J Adolesc. 2001;24:611–24. 10.1006/jado.2000.0393 11676508 10.1006/jado.2000.0393

[28] Schneewind K, Beckmann M, Hecht-Jackl A. Familienklima-Skalen. Bericht 8.1 und 8.2. In:Ludwig Maximilians Universität. München: Institut für Psychologie-Persönlichkeitspsychologie und Psychodiagnostik. 1985.

[29] Donald CA, Ware JE. The measurement of social support. Res Community Ment Health. 1984;4:325–70.

[30] Bauer DJ, Curran PJ. Distributional assumptions of growth mixture models: implications for overextraction of latent trajectory classes. Psychol Methods. 2003;8:338–63. 10.1037/1082-989X.8.3.338. 14596495 10.1037/1082-989X.8.3.338

[31] Lo Y, Mendell NR, Rubin DB. Testing the number of components in a normal mixture. Biometrika. 2001;88:767–78. 10.1093/biomet/88.3.767. 10.1093/biomet/88.3.767

[32] Muthén B. Latent variable analysis: growth mixture modeling and related techniques for longitudinal data. In: Kaplan D, editor. Handbook of quantitative methodology for the social sciences. Newbury Park: Sage; 2004. pp. 345–69.

[33] Muthén B, Muthén LK. Integrating person-centered and variable‐centered analyses: growth mixture modeling with latent trajectory classes. Alcoholism: Clin Exp Res. 2000;24:882–91. 10.1111/j.1530-0277.2000.tb02070.x. 10.1111/j.1530-0277.2000.tb02070.x 10888079

[34] Nesselroade JR. (1991) Interindividual differences in intraindividual change. https://psycnet.apa.org/doi/10.1037/10099-006.

[35] Nylund KL, Asparouhov T, Muthén BO. Deciding on the number of classes in latent class analysis and growth mixture modeling: a Monte Carlo simulation study. Struct Equation Modeling: Multidisciplinary J. 2007;14:535–69. 10.1080/10705510701575396. 10.1080/10705510701575396

[36] Tabachnick B, Fidell LS. Using multivariate statistics. London: Pearson Education Inc.; 2014.

[37] Muthén B, Asparouhov T. Growth mixture modeling: analysis with non-gaussian random effects. In: Fitzmaurice G, Davidian M, Verbeke G, Molenberghs G, editors. Advances in Longitudi-Nal Data Analysis. CRC; 2006. pp. 143–65.

[38] Bullinger M. Quality of life– definition, conceptualization and implications– a methodologists view. Theoretic Surg. 1991;6:143–9.

[39] Cosma A, Abdrakhmanova S, Taut D, Schrijvers K, Catunda C, Schnohr C. (2023) A focus on adolescent mental health and well-being in Europe, central Asia and Canada. In: WHO Regional Office for Europe, editor Health Behaviour in School-aged Children international report from the 2021/2022 survey.

[40] Vanaelst B, De Vriendt T, Ahrens W, Bammann K, Hadjigeorgiou C, Konstabel K, Lissner L, Michels N, Molnar D, Moreno LA. Prevalence of psychosomatic and emotional symptoms in European school-aged children and its relationship with childhood adversities: results from the IDE-FICS study. Eur Child Adolesc Psychiatry. 2012;21:253–65. 10.1007/s00787-012-0258-9. 22350132 10.1007/s00787-012-0258-9

[41] Haller H, Cramer H, Lauche R, Dobos G. Somatoform disorders and medically unexplained symptoms in primary care: a systematic review and meta-analysis of prevalence. Dtsch Arztebl Int. 2015;112:279. 10.3238/2Farztebl.2015.0279 25939319 10.3238/2Farztebl.2015.0279 PMC4442550

[42] Nagin DS, Odgers CL. Group-based trajectory modeling in clinical research. Annu Rev Clin Psychol. 2010;6:109–38. 10.1146/annurev.clinpsy.121208.131413. 20192788 10.1146/annurev.clinpsy.121208.131413

[43] Janousch C, Anyan F, Morote R, Hjemdal O. Resilience patterns of Swiss adolescents before and during the COVID-19 pandemic: a latent transition analysis. Int J Adolesc Youth. 2022;27:294–314. 10.1080/02673843.2022.2091938. 10.1080/02673843.2022.2091938

[44] Landman B, Cohen A, Khoury E, Trebossen V, Bouchlaghem N, Poncet-Kalifa H, Acquaviva E, Lefebvre A, Delorme R. Emotional and behavioral changes in French children during the COVID-19 pandemic: a retrospective study. Sci Rep. 2023. 10.1038/s41598-023-29193-9. 10.1038/s41598-023-29193-9 PMC9897150 36737512

[45] Mitra R, Waygood EOD, Fullan J. Subjective well-being of Canadian children and youth during the COVID-19 pandemic: the role of the social and physical environment and healthy movement behaviours. Prev Med Rep. 2021;23:101404. 10.1016/j.pmedr.2021.101404 34189017 10.1016/j.pmedr.2021.101404 PMC8220397

[46] Orgiles M, Tomczyk S, Amoros-Reche V, Espada JP, Morales A. Stressful life events in children aged 3 to 15 years during the COVID-19 pandemic: a latent class analysis. Psicothema. 2023;35:58–65. 10.7334/psicothema2022.202. 36695851 10.7334/psicothema2022.202

[47] Wang D, Zhao J, Zhai S, Chen H, Liu X, Fan F. Trajectories of mental health status during the early phase pandemic in China: a longitudinal study on adolescents living in the community with confirmed cases. Psychiatry Res. 2022;314:114646. 10.1016/j.psychres.2022.114646 35671562 10.1016/j.psychres.2022.114646 PMC9142367

[48] Bauer DJ, Curran PJ. The integration of continuous and discrete latent variable models: potential problems and promising opportunities. Psychol Methods. 2004;9:3–29. 10.1037/1082-989X.9.1.3. 15053717 10.1037/1082-989X.9.1.3

[49] Newson R. Generalized power calculations for generalized linear models and more. Stata J. 2004;4(4):379–401. 10.1177/1536867X0400400402

[50] Kumle L, Võ ML, Draschkow D. Estimating power in (generalized) linear mixed models: an open introduction and tutorial in R. Behav Res. 2021;53:2528–43. 10.3758/s13428-021-01546-0. 10.3758/s13428-021-01546-0 PMC8613146 33954914

[51] Wirtz M. Rehabilitation (Stuttg). On the problem of missing data: How to identify and reduce the impact of missing data on findings of data analysis. Die Rehabil. 2004;43:109–15. 10.1055/s-2003-814839. 15100920 10.1055/s-2003-814839

