# Supplementary material for: Trajectories of mental health in children and adolescents during the COVID-19 pandemic: findings from the longitudinal COPSY study
Source: Child Adolesc Psychiatry Ment Health. 2024 Jul 18;18:89. doi: 10.1186/s13034-024-00776-2 (PMC11264697; doi:10.1186/s13034-024-00776-2)
Supplement: Supplementary file 1 — Supplementary Material 1 [file 13034_2024_776_MOESM1_ESM.docx]

***Appendix A:* Association of class membership for internalising and externalising mental health problems and psychosomatic symptoms**

***Association of classes: Internalising and externalising mental health problems***

*χ^2^_df=12_ = 389.290; p < .001; Contingency Coefficient: .584*

*Most probable*

*Internalising MHP → Externalising MHP*

*Class 1 resilient → Class 1 resilient (80.0%)*

*Class 2 poor → Class 3 poor and 4 improved (39.7% / 35.6%)*

*Class 3 improved → Class 1 resilient (46.0%)*

*Class 4 improved → Class 3 poor (58.2%)*

|  |  | **Externalising MHP** | | | | |
| --- | --- | --- | --- | --- | --- | --- |
|  |  | **Class 1** | **Class 2** | **Class 3** | **Class 4** | **Class 5** |
| **Internalising MHP** | **Class 1** | 443  80,0% | 46  8.3% | 43  7.8% | 22  4.0% | 0  0.0% |
|  | **Class 2** | 8  11.0% | 6  8.2% | 29  39.7% | 26  35.6% | 4  5.5% |
|  | **Class 3** | 23  46.0% | 13  26.0% | 5  10.0% | 9  18.0% | 0  0.0% |
|  | **Class 4** | 6  9.0% | 0  0.0% | 39  58.2% | 13  19.4% | 9  13.4% |

*Percentages linewise*

***Association of classes: Psychosomatic symptoms and internalising mental health problems***

*χ^2^_df=12_ = 306.782; p < .001; Contingency Coefficient: .540*

*Most probable*

*Psychosomatic symptoms → Internalising MHP*

*Class 1 impaired → Class 1 resilient (85.9%)*

*Class 2 resilient → Class 2 poor (42.6%)*

*Class 3 resilient → Class 1 resilient (54.5%)*

*Class 4 impaired → Class 3 improved (37.5%)*

*Class 5 improved → Class 2 poor (41.7%)*

|  |  | **Internalising MHP** | | | |
| --- | --- | --- | --- | --- | --- |
|  |  | **Class 1** | **Class 2** | **Class 3** | **Class 4** |
| **Psychosomatic symptoms** | **Class 1** | 505  85.9% | 26  4.4% | 29  4.9% | 28  4.8% |
|  | **Class 2** | 13  19.1% | 29  42.6% | 8  11.8% | 18  26.5% |
|  | **Class 3** | 24  54.5% | 3  6.8% | 1  2.3% | 16  36.6% |
|  | **Class 4** | 9  18.1% | 10  31.3% | 12  37.5% | 1  3.1% |
|  | **Class 5** | 3  25.0% | 5  41.7% | 0  0.0% | 4  33.3% |

*Percentages linewise*

***Association of classes: Psychosomatic symptoms and externalising mental health problems***

*χ^2^_df=12_ = 275.51; p < .001; Contingency Coefficient: .520*

*Most probable*

*Psychosomatic symptoms → Externalising MHP*

*Class 1 impaired → Class 1 resilient (75.0%)*

*Class 2 resilient → Class 3 poor and 4 improved (32.4% and 30.9%)*

*Class 3 resilient → Class 3 poor (61.4%)*

*Class 4 impaired → Class 2 improved (40.6%)*

*Class 5 improved → Class 3 poor (33.3%)*

|  |  | **Externalising MHP** | | | | |
| --- | --- | --- | --- | --- | --- | --- |
|  |  | **Class 1** | **Class 2** | **Class 3** | **Class 4** | **Class 5** |
| **Psychosomatic complaints** | **Class 1** | 441  75.0% | 47  8% | 60  10.2% | 38  6.5% | 2  0.3% |
|  | **Class 2** | 16  23.5% | 2  2.9% | 22  32.4% | 21  30.9% | 7  10.3% |
|  | **Class 3** | 13  29.5% | 2  4.5% | 27  61.4% | 1  2.3% | 1  2.3% |
|  | **Class 4** | 7  21.9% | 13  40.6% | 3  9.4% | 8  25.0% | 1  3.1% |
|  | **Class 5** | 3  25.0% | 1  8.3% | 4  33.3% | 2  16.7% | 2  16.7% |

*Percentages linewise*
